# Tibetans exhibit lower hemoglobin concentration and decreased heart response to hypoxia during poikilocapnia at intermediate altitude relative to Han Chinese

**DOI:** 10.3389/fphys.2024.1334874

**Published:** 2024-05-09

**Authors:** E. A. Moya, J. J. Yu, S. Brown, W. Gu, E. S. Lawrence, R. Carlson, A. Brandes, W. Wegeng, K. Amann, S. E. McIntosh, F. L. Powell, T. S. Simonson

**Affiliations:** ^1^ Division of Pulmonary, Critical Care, Sleep Medicine, and Physiology, Department of Medicine, University of California San Diego, La Jolla, CA, United States; ^2^ Department of Anesthesiology, Loyola University Medical Center, Maywood, IL, United States; ^3^ School of Medicine, University of Utah, Salt Lake City, UT, United States; ^4^ Department of Emergency Medicine, University of Rochester Medical Center, Rochester, NY, United States; ^5^ Department of Emergency Medicine, University of Utah Health, Salt Lake City, UT, United States

**Keywords:** high altitude, Tibetan, heart rate, hemoglobin, control of breathing

## Abstract

**Background:**

High-altitude populations exhibit distinct cellular, respiratory, and cardiovascular phenotypes, some of which provide adaptive advantages to hypoxic conditions compared to populations with sea-level ancestry. Studies performed in populations with a history of high-altitude residence, such as Tibetans, support the idea that many of these phenotypes may be shaped by genomic features that have been positively selected for throughout generations. We hypothesize that such traits observed in Tibetans at high altitude also occur in Tibetans living at intermediate altitude, even in the absence of severe sustained hypoxia.

**Methodology:**

We studied individuals of high-altitude ancestry (Tibetans, n = 17 females; n = 12 males) and sea-level ancestry (Han Chinese, n = 6 females; n = 10 males), both who had been living at ∼1300 m (∼4327 ft) for at least 18 months. We measured hemoglobin concentration ([Hb]), hypoxic ventilatory response (HVR), and hypoxic heart rate response (HHRR) with end-tidal CO_2_ (PetCO_2_) held constant (isocapnia) or allowed to decrease with hypoxic hyperventilation (poikilocapnia). We also quantified the contribution of CO_2_ on ventilation and heart rate by calculating the differences of isocapnic *versus* poikilocapnic hypoxic conditions (Δ ${\dot{V}}_{I}$ /ΔPetCO_2_ and ΔHR/ΔPetCO_2_, respectively).

**Results:**

Male Tibetans had lower [Hb] compared to Han Chinese males (*p* < 0.05), consistent with reports for individuals from these populations living at high altitude and sea level. Measurements of ventilation (resting ventilation, HVR, and PetCO_2_) were similar for both groups. Heart rate responses to hypoxia were similar in both groups during isocapnia; however, HHRR in poikilocapnia was reduced in the Tibetan group (*p* < 0.03), and the heart rate response to CO_2_ in hypoxia was lower in Tibetans relative to Han Chinese (*p* < 0.01).

**Conclusion:**

These results suggest that Tibetans living at intermediate altitude have blunted cardiac responses in the context of hypoxia. Hence, only some of the phenotypes observed in Tibetans living at high altitude are observed in Tibetans living at intermediate altitude. Whereas blunted cardiac responses to hypoxia is revealed at intermediate altitudes, manifestation of other physiological adaptations to high altitude may require exposure to more severe levels of hypoxia.

## Introduction

Mammals exposed to low oxygen (O_2_) conditions (hypoxia) maintain O_2_ homeostasis through a variety of physiological responses (Lenfant, 1973; West, 2012). These include both acute responses, such as the hypoxic ventilatory response (HVR) (Teppema and Dahan, 2010; Pamenter and Powell, 2016) and increased heart rate (Richalet and Lhuissier, 2015), as well as time-dependent responses to chronic hypoxia such as ventilatory acclimatization to hypoxia (Powell et al., 1998), increased hematocrit (Beall, 2007), and changes in metabolism (Murray, 2016; O’Brien et al., 2020). Exposure to life-long hypoxia at high altitude in populations throughout many generations may exert evolutionary pressure resulting in adaptations to hypoxia by natural selection. Survival in high-altitude hypoxic environments is possible due to physiological strategies that compensate for low oxygen levels; however, physiological changes may reflect both adaptive and maladaptive responses to this environmental stress (Moore, 2001; Beall, 2006; Beall, 2007; Petousi and Robbins, 2014; Simonson, 2015; Storz and Scott, 2021).

Two populations often studied in terms of responses to hypoxia include Tibetan residents of high altitude (≥3,500 m) and Han Chinese who generally live at low altitudes (Kapoor and Kapoor, 2005; Petousi et al., 2014; Petousi and Robbins, 2014). For example, studies show that pulmonary ventilation is greater in Tibetans than Han Chinese when both are acclimatized to high altitude (Zhuang et al., 1993), and hematocrit is lower in Tibetans compared to Han Chinese living at high altitude (Zhuang et al., 1993; Garruto et al., 2003; Wu et al., 2005; Mairbäurl et al., 2020). While many Tibetans exhibit lower hemoglobin concentration ([Hb]) relative to other individuals at high altitude, many Andean residents develop excessive erythrocytosis, which is associated with chronic mountain sickness (Villafuerte and Corante, 2016; Villafuerte et al., 2022). Hence, lower [Hb] due to alterations in plasma volume (Stembridge et al., 2019) or potential limited erythrocytosis and increased red cell destruction (Tift et al., 2020; Yu et al., 2022) in Tibetans may be adaptive at high altitude.

There is evidence for positive selection in populations that have resided at high altitude for hundreds of generations, including Tibetans (Simonson, 2015; Moore, 2017; Bhandari and Cavalleri, 2019). Recent studies show that many of the same genes are subject to selection across different high-altitude populations, although the specific variants can differ between populations (Simonson, 2015; Bigham, 2016; Witt and Huerta-Sánchez, 2019; Yu et al., 2022; Lawrence et al., 2024), as can the phenotypes (Beall et al., 1997; Beall, 2006; Beall, 2007; Tift et al., 2020; Yu et al., 2022). Several studies have demonstrated strong associations between [Hb] at high altitude and selected genetic loci, e.g., *EPAS1* (Beall et al., 2010; Simonson et al., 2010), although more work is needed to determine whether hemoglobin and hematocrit are the direct target of selection at high altitude or a concomitant change (Storz, 2010; Stembridge et al., 2019; Yu et al., 2022). Research that aims to identify links between [Hb] and other key phenotypes at high altitude may provide insight for future mechanistic studies.

If phenotypic differences observed between populations at high altitude have a genetic basis (Savolainen et al., 2013), it is plausible that such differences may persist independent of hypoxia exposure or demonstrate distinct gene-by-environment effects. Petousi et al. (2014) studied Tibetan individuals born at high altitude but living near sea level and compared them with Han Chinese individuals living in the same location. They found significantly lower [Hb] and higher ventilation (relative to metabolism) in Tibetans (Petousi et al., 2014), which is consistent with results collected in these groups residing at high altitude (Zhuang et al., 1993). However, in contrast to studies at high altitude comparing Tibetan to Han Chinese and other populations (Zhuang et al., 1993; Beall, 2000; Beall, 2006; Beall, 2007), they found no differences in the hypoxic ventilatory response (HVR) between Tibetans and Han Chinese. Unlike [Hb] and ventilation, the heart rate response to hypoxia has not been extensively studied in Tibetans. Blunted heart rate responses to hypoxia were observed in Tibetan individuals who, compared to participants with an ancestral history of sea-level residence, traveled from sea level to high altitude (Reeves et al., 1987; Groves et al., 1993; Bärtsch and Gibbs, 2007; Gonggalanzi et al., 2017); however, differences in acute normobaric hypoxic heart rate response in individuals with high-altitude and sea-level ancestries has not been demonstrated. Our previous research conducted on Andeans residing at an altitude of 4340 m identified an association between increased hematocrit (a measure of red blood cell concentration) and decreased hypoxic heart rate response (HHRR), suggesting a relationship between these oxygen transport components in highland populations (Heinrich et al., 2020). We extend such observations to examine individuals of Tibetan and Han Chinese ancestry living at an intermediate altitude (1,319 m) and test for differences in heart rate responses to hypoxia.

Our ultimate goals are to determine whether sensitivity to oxygen varies between individuals with high-altitude and sea-level ancestries in different environmental contexts, investigate how these phenotypes relate to other components of oxygen transport, and use this information to assess the genetic basis for phenotypic variation over a range of altitudes. We hypothesize that Tibetans living at intermediate altitude show traits of adaptation observed in high-altitude populations when compared to a Han Chinese control group. We measured [Hb], as well as ventilatory and heart rate responses to acute hypoxia, in Tibetans and Han Chinese residents of intermediate altitude and explored the relationships between these measuements.

## Methods

### Ethical consideration

This study was approved by the University of San Diego Human Research Protections Program (#140235). Measurements were completed at the University of Utah, School of Medicine (1,319 m above sea level).

### Participants

All participants of Han Chinese (6 female and 10 male) and Tibetan (17 female and 12 male) ancestries resided in Salt Lake City, UT, for a minimum of 6 months, which exceeds current estimates of red blood cell lifespan and time needed for ventilatory de-acclimatization from chronic hypoxia exposure. The exclusion criteria included travel to altitudes above 2,500 m in the previous month, a history of cardiovascular or pulmonary disease, as well as participants with past or current history of smoking. Participants had self-reported Tibetan or Han Chinese ancestry history. Two Han Chinese and nine Tibetan participants did not provide blood samples for measurement of hemoglobin concentration. For measurements of ventilation and heart rate responses to hypoxia, six female and 10 male Han Chinese and 13 female and 10 male Tibetan performed the experiments. Two Tibetan males were excluded from the physiology portion of the study as their SpO_2_ did not decrease the full 10% during hypoxia required for this study. One Tibetan women did not complete ventilatory studies and another had measurements of isocapnia but not poikilocapnia. One Han Chinese and three Tibetan post-menopausal women were included in the study. All participants provided written consent to documents provided in English. Medical histories and physical examinations were performed during a preliminary screening session to verify no prior history of cardiovascular or pulmonary disease and current use of interfering medications. During this period, participants were also questioned about their history of high-altitude residence and ancestral background (self-identified ancestry and geographical location of their parents and grandparents). We measured height, weight, BMI, as well as systolic (SBP) and diastolic (DBP) blood pressure using a manual sphygmomanometer. Mean arterial blood pressure (MABP) was calculated as MABP = DBP + (1/3)*(SBP - DBP). Participants did not consume caffeine for at least 8 h and corticosteroids nor NSAIDs for at least 48 h prior to physiological tests.

### Hemoglobin concentration

[Hb] was measured via AVOXimeter 4000 (Avox Systems, Inc., San Antonio, TX) immediately following venous blood draw.

### Hypoxic response measurements

Participants completed an 8–10-min abbreviated version of the full ventilatory response protocol to allow for acclimation to the devices and to determine appropriate individual gas flows needed to reach their target oxygen saturations (SpO_2_) and end-tidal P _CO2_ (PetCO_2_). Participants returned after a >30-min rest period to complete the full protocol. This rest period and preparation for the next test allowed sufficient time for recovery from hypoxic ventilatory decline induced by the screening session hypoxia exposure (Easton et al., 1988). During testing, participants sat in a chair and a mask was placed over the mouth and nose (7600 V2 Oro-Nasal Mask, Hans Rudolph, Shawnee, KS); leaks were checked by having the participant inhale against a closed inspiratory valve to ensure a vacuum was produced. The mask was connected to a one-way vented breathing circuit with a non-rebreathing valve (2700 Series, Large, Hans Rudolph). A three-way valve upstream of the mask allowed either room air or gas mixtures of N_2_/O_2_/CO_2_ to flow into the circuit. O_2_ and CO_2_ were continuously sampled from the tube right before the mask or non-rebreathing valve directly in front of the mouth, respectively (Model 17,620 and 17515A, VacuMed, Ventura, CA, United States). Inspiratory flow was measured by a pneumotachograph (Fleisch No. 3, OEM Medical Inc., Richmond, VA, United States of America) upstream of the mask and connected to a differential pressure transducer (model CD15, Valine, Northridge, CA, United States of America). Sp_O2_ and heart rate were measured by a pulse oximeter (Radical-7, Masimo Corporation Irvine, CA, United States) with a surface probe placed on the forehead. All analog signals were processed through a PowerLab 8/30 (ADInstruments, Colorado Springs, CO, United States) and sent digitally to a laptop computer (HP Probook, HP Inc., Palo Alto, CA, United States). Raw data was recorded in LabChart 8 (ADInstruments).

Gas mixtures were manually controlled with a three-channel rotameter flow meter (Matheson Gas Products, Montgomeryville, PA, United States) that delivered mixtures upstream of the three-way valve at flow rates sufficient to prevent rebreathing. We measured Sp_O2_, fraction of inspired O_2_ (Fi_O2_), fraction of inspired CO_2_ (Fi_CO2_), tidal volume (V_T_), respiratory frequency (f_R_), minute ventilation ( ${\dot{V}}_{I}$ ), PetCO_2_, and heart rate (HR) continuously during the protocol. Participants breathed ambient air for 3–5 min followed by 5 min of mild hyperoxia (∼230 mmHg, simulating 30% Fi_O2_ at sea level). This hyperoxic phase was required to reverse potential hypoxic ventilatory decline in these participants, as applied in previous studies with people exposed to continuous hypoxemia at high altitude. To determine the HVR in isocapnic conditions, participants then breathed a hypoxic gas mixture during 5 min as we targeted 3 min of stable Sp_O2_ that produced a decrease of at least 10% from the levels recorded during previous hyperoxic measurements (Heinrich et al., 2020). Isocapnia was achieved throughout the hypoxic phase by manually adding CO_2_ to maintain the average PetCO_2_ value observed during the last minute of the previous hyperoxic period. Isocapnic hypoxic events with PetCO_2_ more than 2 mmHg different than the PetCO_2_ observed during previous hyperoxia within the same participant were removed (Figure 2A) (two male Tibetan participants were excluded from the HVR analysis based on this exclusion criteria). Participants were then exposed to 5 min of hyperoxia (30% Fi_O2_ at sea level) followed by 5 minutes of poikilocapnic hypoxia, where we again targeted a 10% decrease in Sp_O2_ but this time without adding CO_2_ to the mixture, allowing changes in PetCO_2_ (Figure 2B). The isocapnic HVR and poikilocapnic HVR were calculated as the change in ventilation per decrease in Sp_O2_ (ΔV.i / ΔSp_O2_) based on the average V.i values obtained during a minimum period of 30 s during hyperoxic and then hypoxic conditions (marked in gray in Figure 2). Since hyperventilation induced by hypoxia produces a decrease in the levels of PetCO_2_ (in poikilocapnia), we calculated the ventilatory response to changes in CO_2_ during hypoxia (HCVR) by measuring the change in ventilation per mmHg decrease in PetCO_2_ (ΔV.i/Δ PetCO_2_) during a minimum period of 30 s, comparing isocapnic *versus* poikilocapnic hypoxia. The hypoxic heart rate response (HHRR) was calculated as the change in heart rate per decrease in Sp_O2_ during hypoxia and hyperoxic treatment steps (ΔHR/Δ Sp_O2_), and the heart rate response to changes in CO_2_ during hypoxia (HHRCR) was calculated as the change in heart rate per mmHg decrease in PetCO_2_ (ΔHR/-Δ PetCO_2_) between isocapnic and poikilocapnic hypoxia.

### Statistical analysis

Data are expressed as mean ± standard error. Statistical analyses comparing two groups were performed using Students t-tests between groups of individuals with Han Chinese and Tibetan ancestries. We performed two-way ANOVA analyses to test ancestry and sex differences within measurements and three-way ANOVA analyses to test the effects of ancestry, sex, and hypoxia exposure on the different variables. The results show significance of both ancestry and sex denoted by ψ and ɸ, respectively. Sidak *post hoc* test was performed when appropriate, and *p* < 0.05 was considered statistically significant for all tests.

We quantified significant correlations between measurements with Pearson correlation coefficients. We also determined whether slopes and intercepts were significantly different from comparisons of simple linear regressions generated for each group. For all statistical analysis, we used GraphPad Prism version 9.0.2 for Windows, GraphPad Software, San Diego, California, United States.

## Results

### Hemoglobin concentration

**FIGURE 1:**
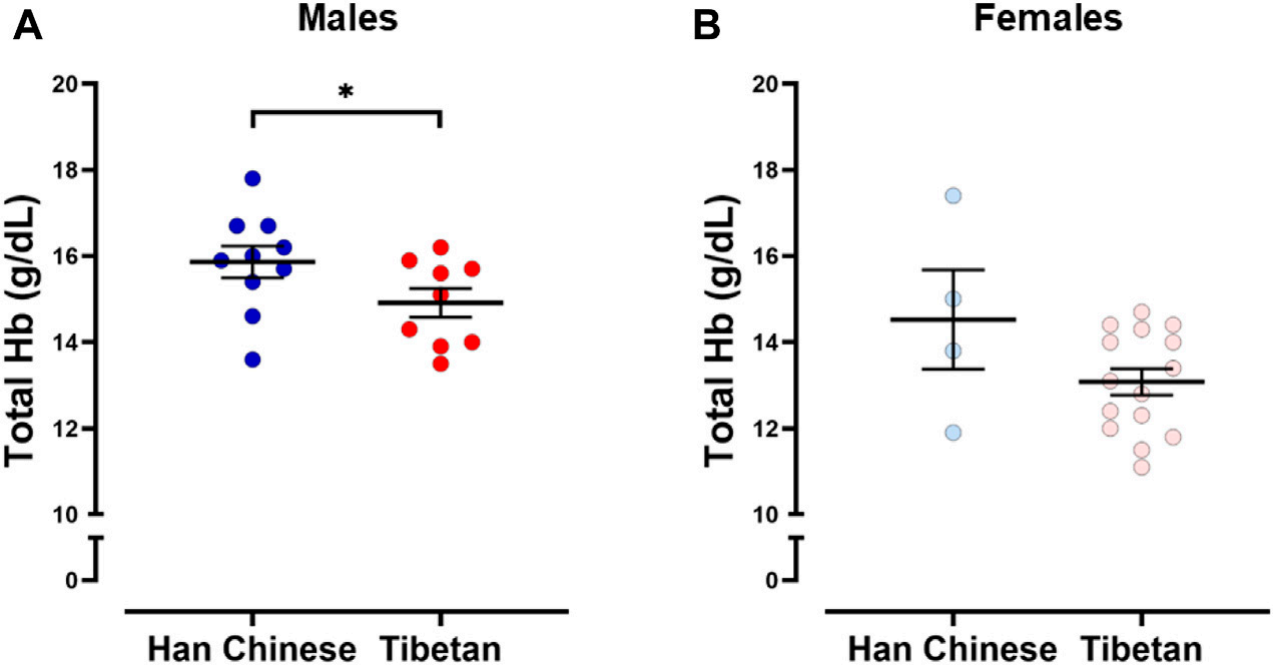
Hemoglobin concentration ([Hb]) in individuals with Tibetan and Han Chinese ancestry. We observed lower [Hb] in male Tibetans (red) compared to participants of Han Chinese (blue) ancestry **(A)**, One-tailed Student’s t-test, *p < 0.05 (Han Chinese n = 10; Tibetan n = 9). Similar analysis performed in Females **(B)** did not show significant differences (*p* = 0.151 Han Chinese n = 4; Tibetan n = 15).

The analysis grouping together males and females revealed that Tibetans have significantly lower [Hb] (13.8 ± 0.29 g/dL) compared to participants of Han Chinese ancestry (15.5 ± 0.43 g/dL, Student’s t-test, *p* < 0.003). Since sex has an effect on [Hb] at high altitude (Beall, 2007), we performed analysis grouping by sex. We found significantly higher values of [Hb] in Han Chinese compared to Tibetan males (15.9 ± 0.34 g/dL and 14.9 ± 0.33 g/dL, respectively, *p* < 0.05, Figure 1A). The analysis performed in females did not show significant differences (*p* = 0.151, 14.53 ± 1.15 g/dL and 13.08 ± 0.31 g/dL for Han Chinese and Tibetan women, respectively, Figure 1B).

### Physiological measurements in normoxia

A summary of demographic variables and physiological measurements recorded during baseline room air conditions is provided in Table 1. The average age of participants was not different between groups. We observed a significant effect of sex on height (*p* < 0.0001) and weight (*p* < 0.0001) between groups, although sex had no effect on BMI. Post-hoc analysis revealed significantly lower values of height and weight for women compared to men within the same ancestry group (Table 1).

**TABLE 1:** Physiological parameters during baseline room air conditions. Mean ± EE.

Parameter	Han Chinese			Tibetan		ANOVA *P*
	Male (10)	Female (6)		Male (8)	Female (13)	
Age (years)	31 ± 4.1 (10)	30.5 ± 6.8 (6)		35.6 ± 6.1 (8)	37.6 ± 4.8 (13)	n.s.
Height (m)	1.77 ± 0.01 (9)	1.63 ± 0.03 (5)*		1.75 ± 0.02 (8)	1.61 ± 0.01 (13)*	**ɸ**
Weight (kg)	71.7 ± 2.3 (10)	56.2 ± 2.7 (6)*		76.2 ± 2.7 (8)	64.1 ± 3.3 (13)*	**ɸ**
BMI (kg/m^2^)	22.7 ± 1.0 (9)	20.8 ± 1.0 (5)		25.1 ± 1.2 (8)	24.8 ± 1.2 (13)	**ψ**
V_T_ (L/kg)	0.0114 ± 0.0006 (10)	0.0092 ± 0.0011 (6)		0.0100 ± 0.0011 (8)	0.0094 ± 0.0009 (13)	n.s.
f_R_ (BrPM)	14 ± 1 (10)	18 ± 2 (6)		18 ± 2 (8)	17 ± 1 (13)	n.s.
${\dot{V}}_{I}$ (L/min*kg)	0.158 ± 0.007 (10)	0.152 ± 0.009 (6)		0.175 ± 0.015 (8)	0.159 ± 0.015 (13)	n.s.
PetCO_2_ (mmHg)	37.2 ± 0.6 (10)	34.2 ± 0.6 (6)		35.0 ± 1.2 (8)	35.4 ± 0.8 (13)	n.s.
HR (BPM)	71 ± 2 (10)	70 ± 1 (6)		68 ± 4 (8)	72 ± 3 (13)	n.s.
Sp_O2_, (%)	96.6 ± 0.4 (10)	96.4 ± 0.6 (6)		96.9 ± 0.7 (8)	98.0 ± 0.3 (13)	**ψ**
MABP (mmHg)	91.1 ± 3.0 (9)	82.7 ± 1.7 (6)		94.5 ± 2.0 (7)	90.4 ± 4.6 (13)	n.s.
SBP (mmHg)	120.4 ± 3.0 (9)	113.3 ± 2.6 (6)		122.3 ± 2.4 (7)	121.1 ± 5.4 (13)	n.s.
DBP (mmHg)	76.4 ± 3.9 (9)	67.3 ± 1.5 (6)		80.6 ± 2.3 (7)	75.2 ± 4.7 (13)	n.s.

Two-way ANOVA, ɸ significant effect of Sex, ψ significant effect of Ancestry, *p* < 0.05.

Post Hoc Analysis, * significant effect male vs female with the same ancestry, *p* < 0.05.

The two-way ANOVA showed a significant effect of ancestry on BMI and Sp_O2_ while breathing room air (*p* < 0.02 for BMI and *p* < 0.047 for Sp_O2_, Table 1); however, *post hoc* analysis did not reveal significant differences between groups (*p* > 0.05). We did not find significant differences for physiological features such as ventilatory parameters (V_T_, f_R_ and V.i), heart rate, and blood pressure measurements in participants breathing room air (Table 1).

### Hypoxic ventilatory and heart rate responses

**FIGURE 2:**
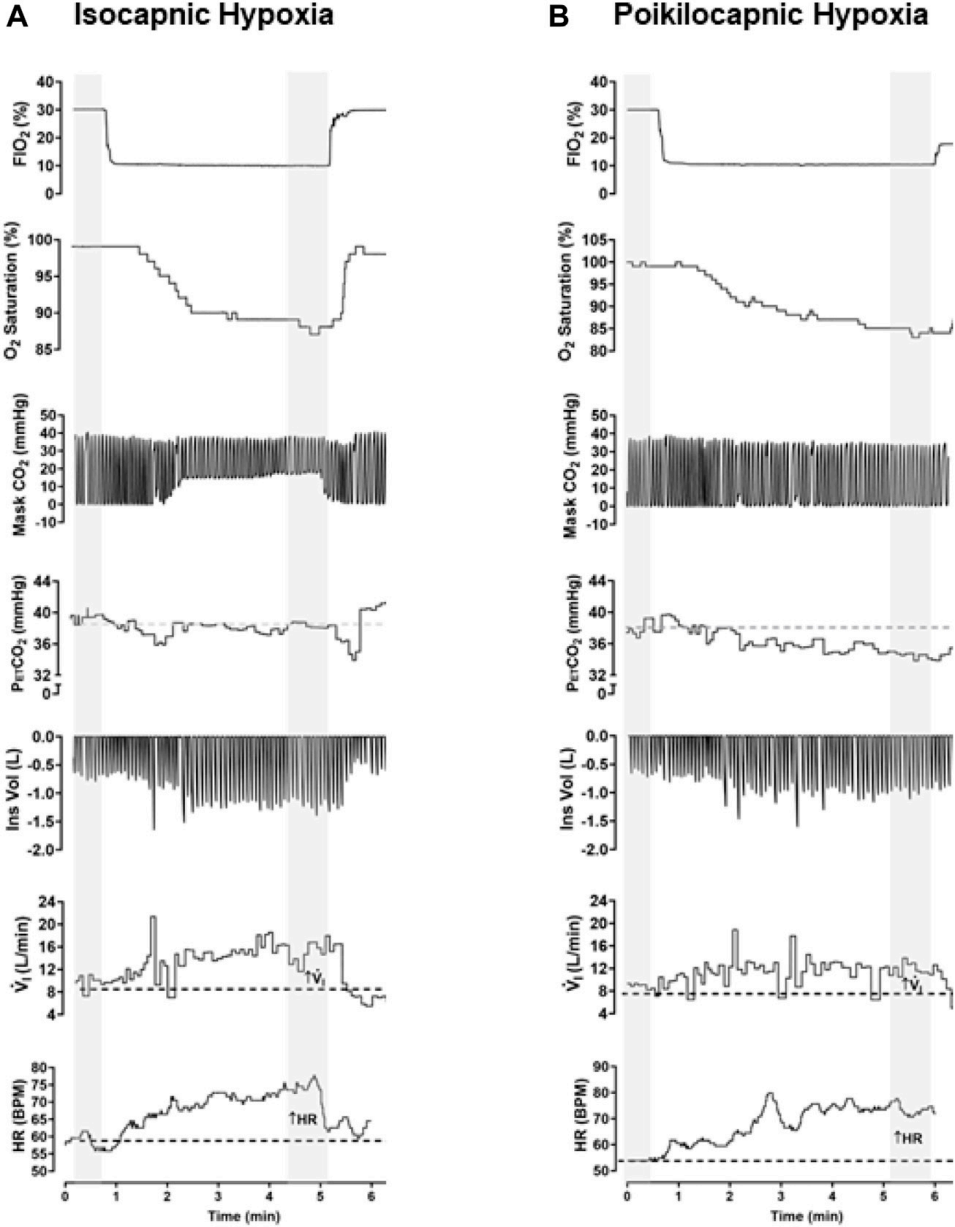
Example of raw data recorded in the same participant in conditions of isocapnic **(A)** and poikilocapnic hypoxia **(B)**. Simultaneous recordings of fraction of inspired oxygen (Fi_O2_), O_2_ saturation (Sp_O2_), concentration of CO_2_ in the mask of the participant (Mask CO_2_), end-tidal P _CO2_ (PetCO_2_) inspiratory volume (Ins Vol), minute ventilation (V.i), and heart rate (HR). For analysis, a minimum of 30 s of recording was averaged during baseline and stable signals during hypoxic episodes (marked in gray).

Figure 2 shows simultaneous example recordings used to measure the hypoxic ventilatory and heart rate responses during conditions of isocapnia and poikilocapnia.

**FIGURE 3:**
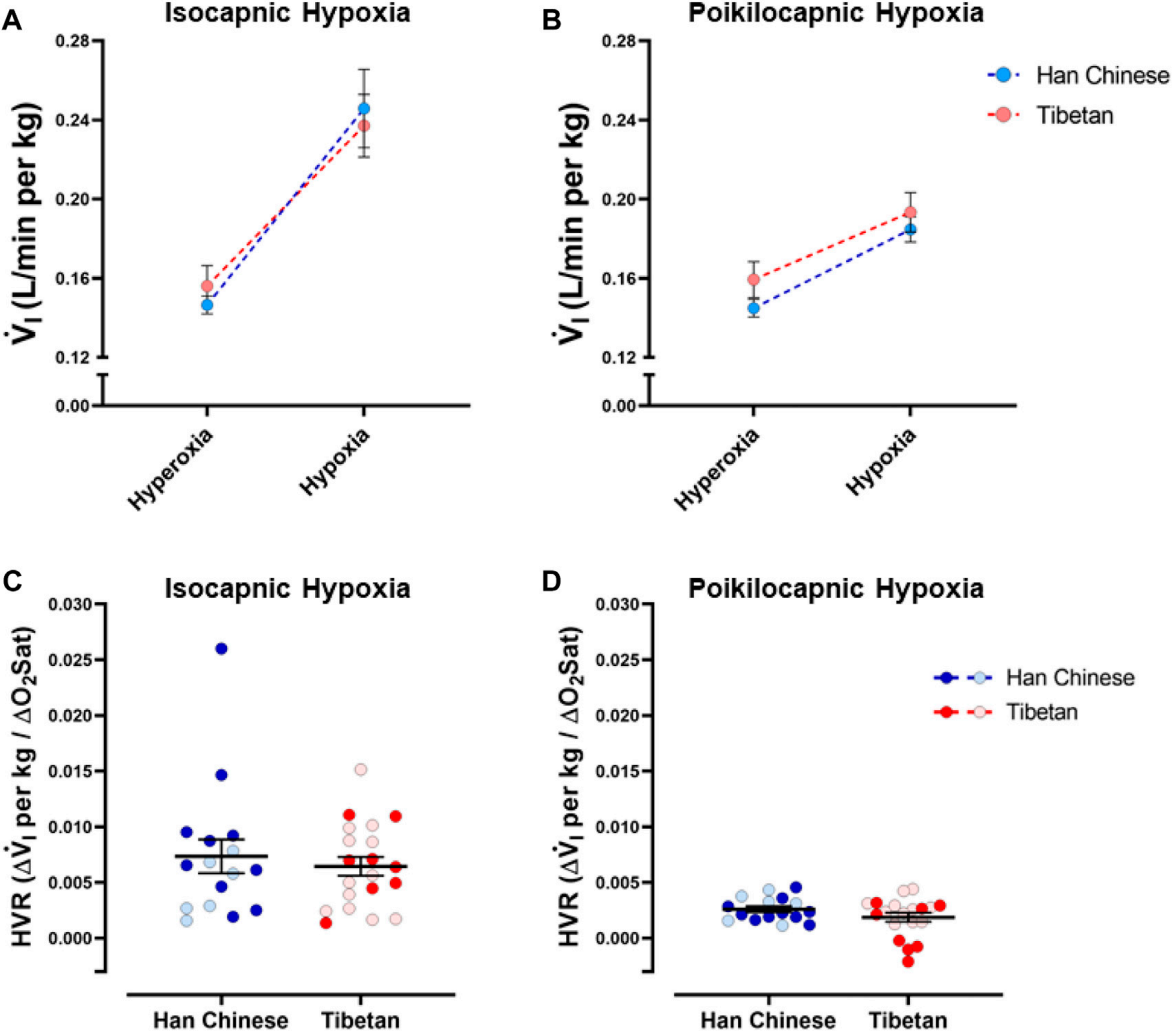
Ventilatory response to hypoxia. Measurements of minute ventilation (V.i) and hypoxic ventilatory responses (HVR) in conditions of isocapnia **(A,C)** and poikilocapnia **(B,D)** in Tibetan (red) and Han Chinese (blue) individuals. Two-way ANOVA performed for isocapnic and poikilocapnic conditions in figures **(A,C)** reveal a significant effect of acute hypoxia (*p* < 0.0001), but no effects of ancestry. Analysis of HVR in **(C,D)** did not show significant differences. Han Chinese n = 16 (10 males, 6 females); Tibetans n = 20 in isocapnia (8 males, 12 females) and 19 in poikilocapnia (8 males, 11 females). For **(C,D)** males in dark blue and red circles and female in light blue and red circles.

As expected, we found a significant effect of hypoxia on V.i for both isocapnic (Figure 3A, *p* < 0.0001) and poikilocapnic hypoxia (Figure 3B, *p* < 0.0001); however, ancestry was not associated with significant differences in V.i. Furthermore, we did not find significant differences in HVR between Tibetan and Han Chinese ancestry for isocapnic (Figure 3C) nor poikilocapnic hypoxia (Figure 3D). No significant effect of sex was revealed with V.i, although there was a significant interaction between ancestry, sex, and hypoxia for poikilocapnic hypoxia (*p* < 0.04, three-way ANOVA). We did not find a significant effect of ancestry in isocapnic or poikilocapnic conditions of HVR, although we did find a significant effect of sex (*p* < 0.03, two-way ANOVA) for poikilocapnic hypoxia. Post-hoc analysis of poikilocapnic hypoxia demonstrated non-significant differences in the Han Chinese group due to sex but higher values of HVR in Tibetan women (0.0026 ± 0.0003 L/min∙%) *versus* Tibetan men (0.0008 ± 0.0007 L/min∙%) (*p* < 0.05).

**FIGURE 4:**
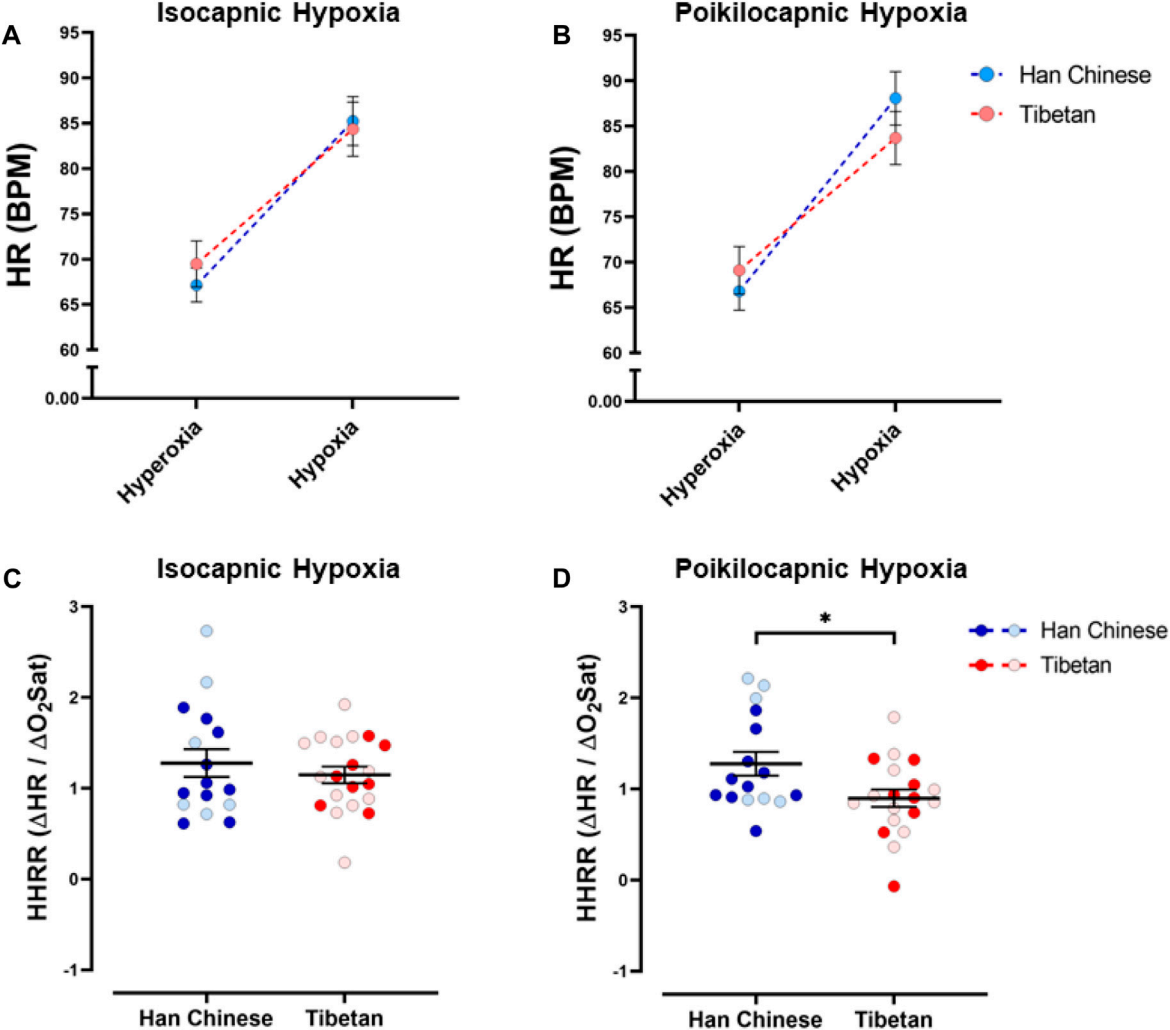
Heart rate responses to hypoxia. Measurements of heart rate (HR) and hypoxic heart rate response (HHRR) in conditions of isocapnia **(A,C)** and poikilocapnia **(B,D)** in Tibetan (red) and Han Chinese (blue) individuals. Two-way ANOVA performed for isocapnic and poikilocapnic conditions for HR in figures **(A,B)** reveal a significant effect of acute hypoxia (*p* < 0.0001), but no independent effects of ancestry. There is a significant interaction between hypoxia and ancestry status for poikilocapnic hypoxia (*p* < 0.05). Student’s t-test analysis of HHRR in isocapnic hypoxia **(C)** did not show significant differences; however, we found a significant effect of ancestry in poikilocapnic hypoxia (**(D)**, *p < 0.05). Two-way ANOVA analysis confirmed the effects of ancestry and revealed no effect of sex. Han Chinese n = 16 (10 males, 6 females); Tibetans n = 20 in isocapnia (8 males, 12 females) and 19 in poikilocapnia (8 males, 11 females). For **(C,D)** males in dark blue and red circles and female in light blue and red circles.

We found a significant effect of hypoxia on heart rate (HR) (*p* < 0.0001), but ancestry did not have a significant effect in isocapnic (Figure 4A) nor poikilocapnic hypoxia (Figure 4B). We identified an interaction of hypoxia and ancestry during poikilocapnic hypoxia (*p* < 0.03) with no sex difference.

Quantification of the HHRR showed no significant differences during isocapnic hypoxia (Figure 4C). However, Tibetans have HHRR values significantly lower than those observed in the Han Chinese group during poikilocapnic hypoxia (Figure 4D; Han Chinese 1.28 ± 0.13 BPM/% Sp_O2_ vs Tibetans 0.90 ± 0.10 BPM/% Sp_O2_; *p* < 0.027), with confirmed effects of ancestry (*p* < 0.012, two-way ANOVA), although we did not observe significant effects of sex on HHRR.

### Ventilatory and heart rate responses to changes in CO_2_ during hypoxia

**FIGURE 5:**
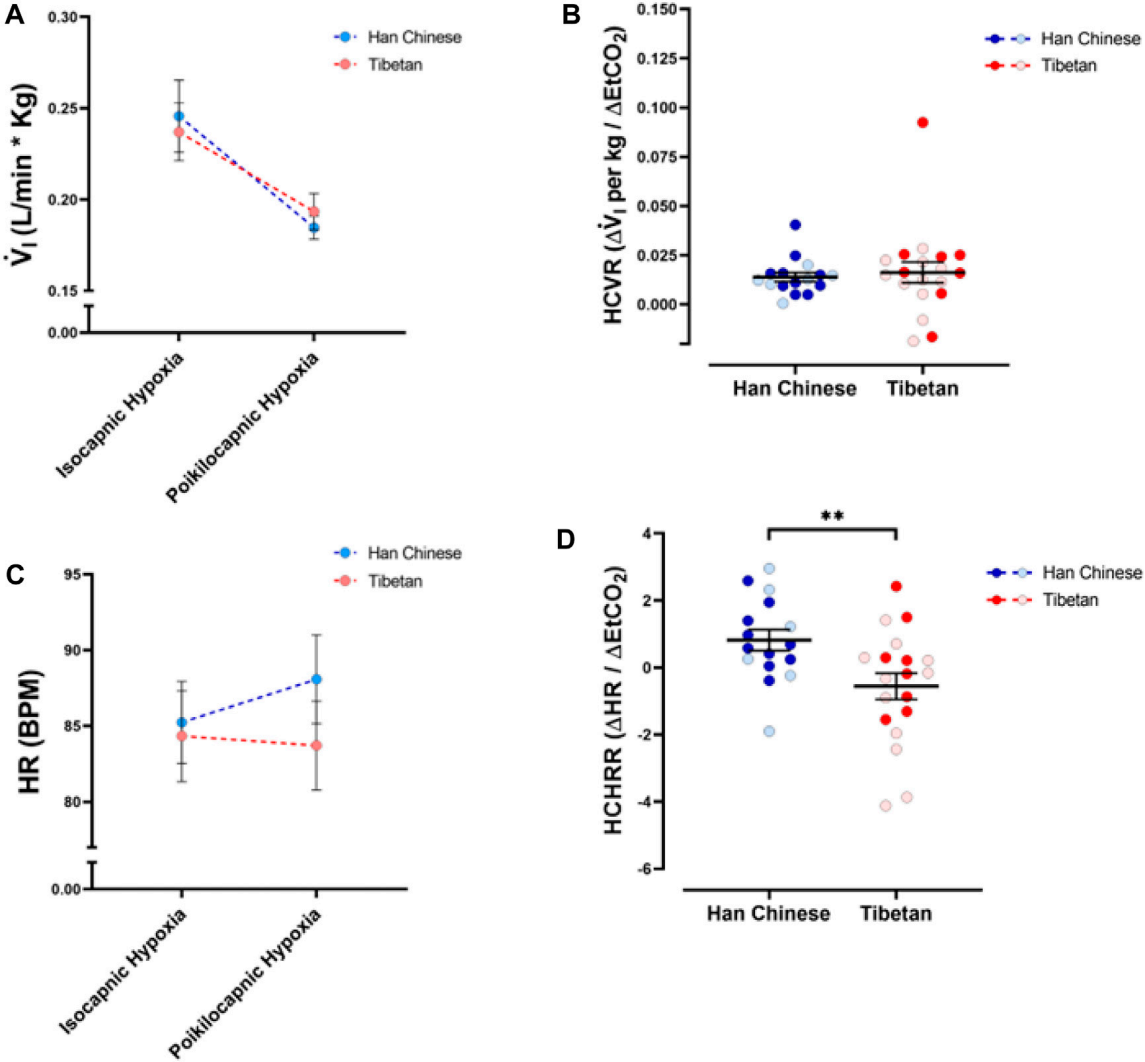
Ventilatory and heart rate responses to changes of CO_2_ during hypoxia. Measurements of minute ventilation (V^.^ i, **(A)**) and heart rate (HR, **(C)**) comparing values during isocapnic and poikilocapnic hypoxia. Measurements of the differences in isocapnic and poikilocapnic hypoxia for ventilation (HCVR, **(B)**) and heart rate (HCHRR, **(D)**) are shown for males (dark circles) and females (light circles) individuals of Tibetan (red) and Han Chinese (blue) ancestry. Two-way ANOVA performed for V^.^ i **(A)** showed a significant effect of changes in CO_2_ during hypoxia (poikilocapnia *versus* isocapnia, *p* < 0.0001) but no effects of ancestry. Similar analysis of HR **(C)** did not show significant effects of ancestry or CO_2_ levels; however, there is a significant interaction of the two factors (*p* < 0.01). Analysis of HCVR did not show statistical differences **(B)**; however, we found a significant effect of ancestry for HCHRR (**(D)**, **p < 0.01). Han Chinese n = 16 (10 males, 6 females); Tibetans n = 19 (8 males, 11 females). For **(B,D)** males in dark colors and female in light colors.

To measure the sensitivity to changes in CO_2_, we quantified differences in V.i and HR between isocapnic and poikilocapnic hypoxia. The different levels of CO_2_ in these two conditions have a significant effect on V.i ( Figure 5A) and, although sex did not have a significant effect itself, there is a significant interaction of CO_2_ and sex over V.i (*p* < 0.05). When we compared HR in these two conditions, we did not find significant effects of CO_2_ levels, nor ancestry (Figure 5C), although there was a significant interaction between the two factors (*p* < 0.006). Additional analysis to account for sex did not show significant differences.

We also measured the hypercapnic ventilatory response (hypoxic HCVR) and the hypercapnic heart rate response (hypoxic HCHRR) by comparing the values of V.i and HR between isocapnic and poikilocapnic conditions during the hypoxic stimulus. We did not find any differences in the hypoxic HCVR between Tibetan and Han Chinese groups (Figure 5B), and sex did not account for any differences. However, we found significant differences in the hypoxic HCHRR (*p* < 0.01) between Han Chinese and Tibetan groups. Participants with Han Chinese ancestry exhibited mean positive values of HCHRR (0.82 ± 0.31 BPM/mmHg) whereas Tibetans show a mean negative HCHRR (−0.56 ± 0.39, Figure 5D). Thus, on average, the Han Chinese group exhibited a greater heart rate response with poikilocapnia *versus* isocapnia, whereas the magnitude of the increase in HR induced by hypoxia is smaller in poikilocapnic compared to isocapnic conditions in the Tibetan group (Figure 5C). Analysis of HCHRR confirmed the significant effect of ancestry (*p* = 0.02, two-way ANOVA) but did not show a significant effect of sex.

**FIGURE 6:**
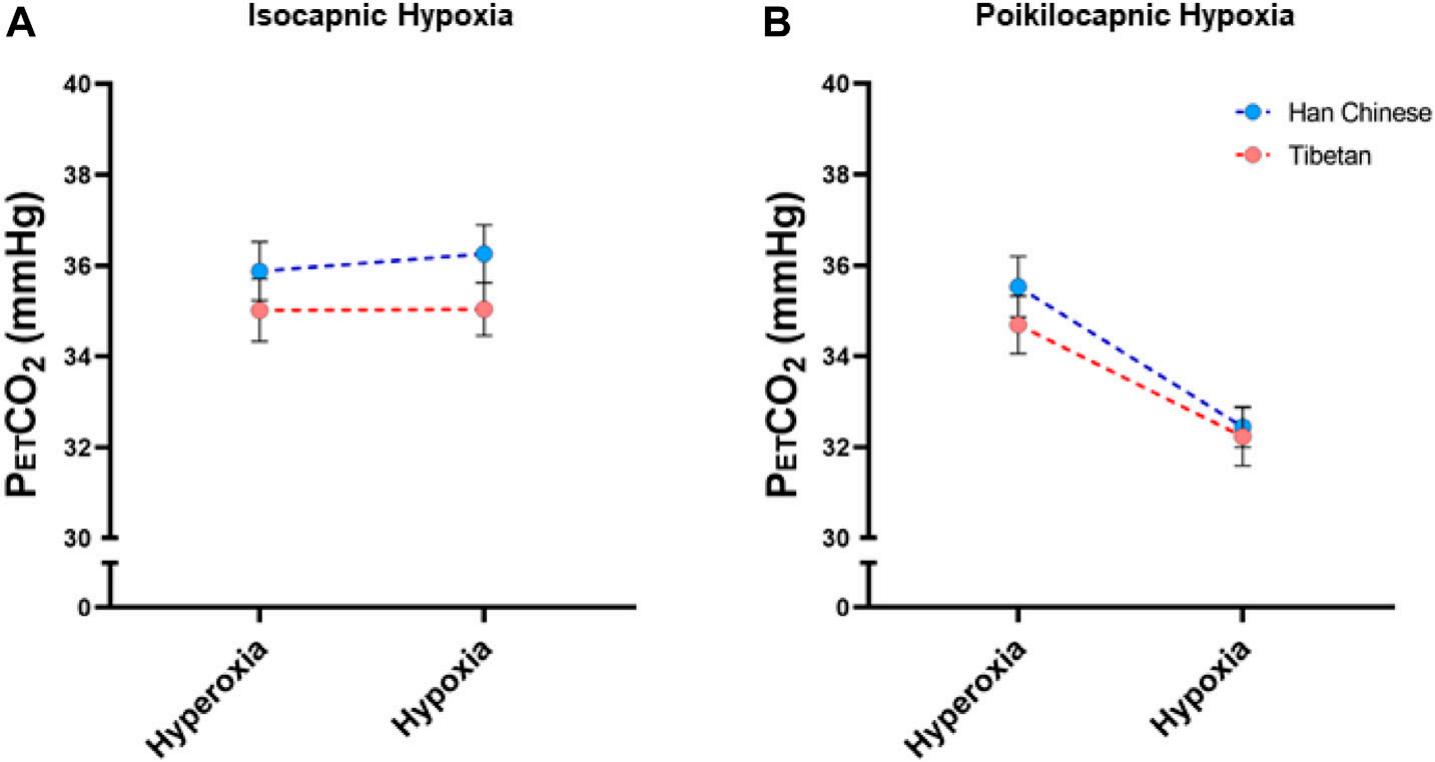
End-tidal CO_2_ measurements (PetCO_2_) measured in conditions of isocapnia **(A)** and poikilocapnia **(B)** in Tibetan (red) and Han Chinese (blue), male (dark circles) and female (light circles) individuals. Analysis of PetCO_2_ in isocapnic hypoxia **(A)** did not show significant differences, and we found a significant effect of acute hypoxia (*p* < 0.0001) in poikilocapnic hypoxia **(B)** with no effects of ancestry. Han Chinese n = 16; Tibetans n = 20 in isocapnia and 19 in poikilocapnia.

We measured PetCO_2_ in isocapnic and poikilocapnic hypoxia (Figure 6). As expected, acute hypoxia produced a significant decrease in Sp_O2_ in both isocapnic and poikilocapnic conditions (*p* < 0.0001 for both, data not shown) without significant effects of ancestry or sex. There was a significant effect of hypoxia on PetCO_2_ in poikilocapnic hypoxia (Figure 6B, *p* < 0.0001) but no effect of hypoxia in isocapnic conditions due to the intentional addition of CO_2_ to maintain baseline PetCO_2_ values (Figure 6A). We did not find significant differences due to ancestry in any of the conditions for levels of PetCO_2_.

### Relationships between HVR and HHRR

**FIGURE 7:**
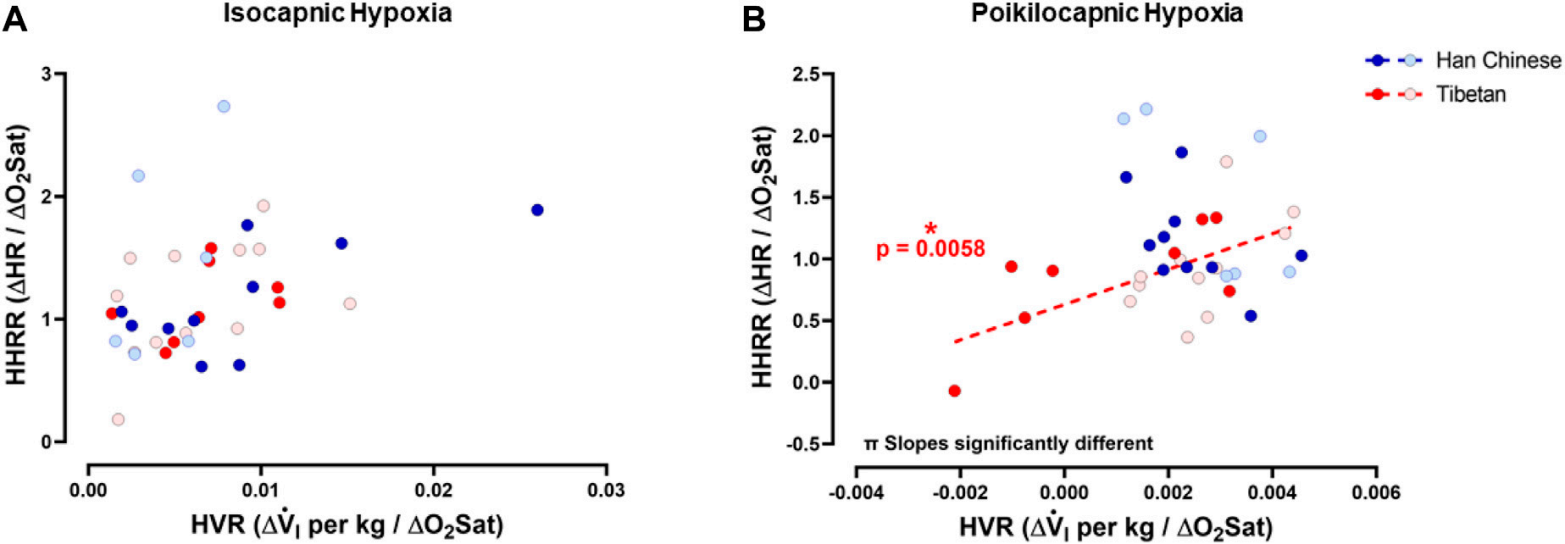
Hypoxic ventilatory response (HVR) *versus* hypoxic heart rate response (HHRR). Relationships between HVR and HHRR during isocapnic **(A)** and poikilocapnic hypoxia **(B)** in Tibetan (red) and Han Chinese (blue), male (dark circles) and female (light circles) individuals. * Significant correlation for Tibetans (red asterisk); π *p* < 0.05 comparing slopes for linear regression of Tibetan *versus* Han Chinese groups. Han Chinese n = 16 (10 males, 6 females); Tibetans n = 20 in isocapnia (8 males, 12 females) and 19 in poikilocapnia (8 males, 11 females).

To test if hypoxia produced a coordinated ventilatory and heart rate response, and for possible interactions between ventilatory and heart rate responses during hypoxia, we examined the relationships between HVR and HHRR under isocapnic and/or poikilocapnic conditions. We did not find significant correlations between HVR and HHRR for Han Chinese nor Tibetan groups during isocapnic hypoxia, and the linear correlations derived from those measurements were not different between groups (Figure 7A). We observed a significant positive correlation of HVR and HHRR for the Tibetan group during poikilocapnic conditions (*p* < 0.006) (Figure 6B), and the slopes of the linear correlations between HVR and HHRR were significantly different when we compared Han Chinese and Tibetans (*p* < 0.002) during poikilocapnic hypoxia (Figure 7B). When we explored the associations of HVR and HHRR separating by sex, we found Han Chinese males exhibited a significant correlation in isocapnic hypoxia (*p* < 0.03), which was not observed in Han Chinese females nor in Tibetan males and females. In poikilocapnic hypoxia, we found a significant correlation between HVR and HHRR in Tibetan males (*p* < 0.04) but not for Han Chinese males nor any of the groups of females. We found significantly different slopes of HVR *versus* HHRR in both Han Chinese and Tibetan males (*p* < 0.02) and females (*p* < 0.02) during poikilocapnic hypoxia.

### HVR and HHRR relative to [Hb]

**FIGURE 8:**
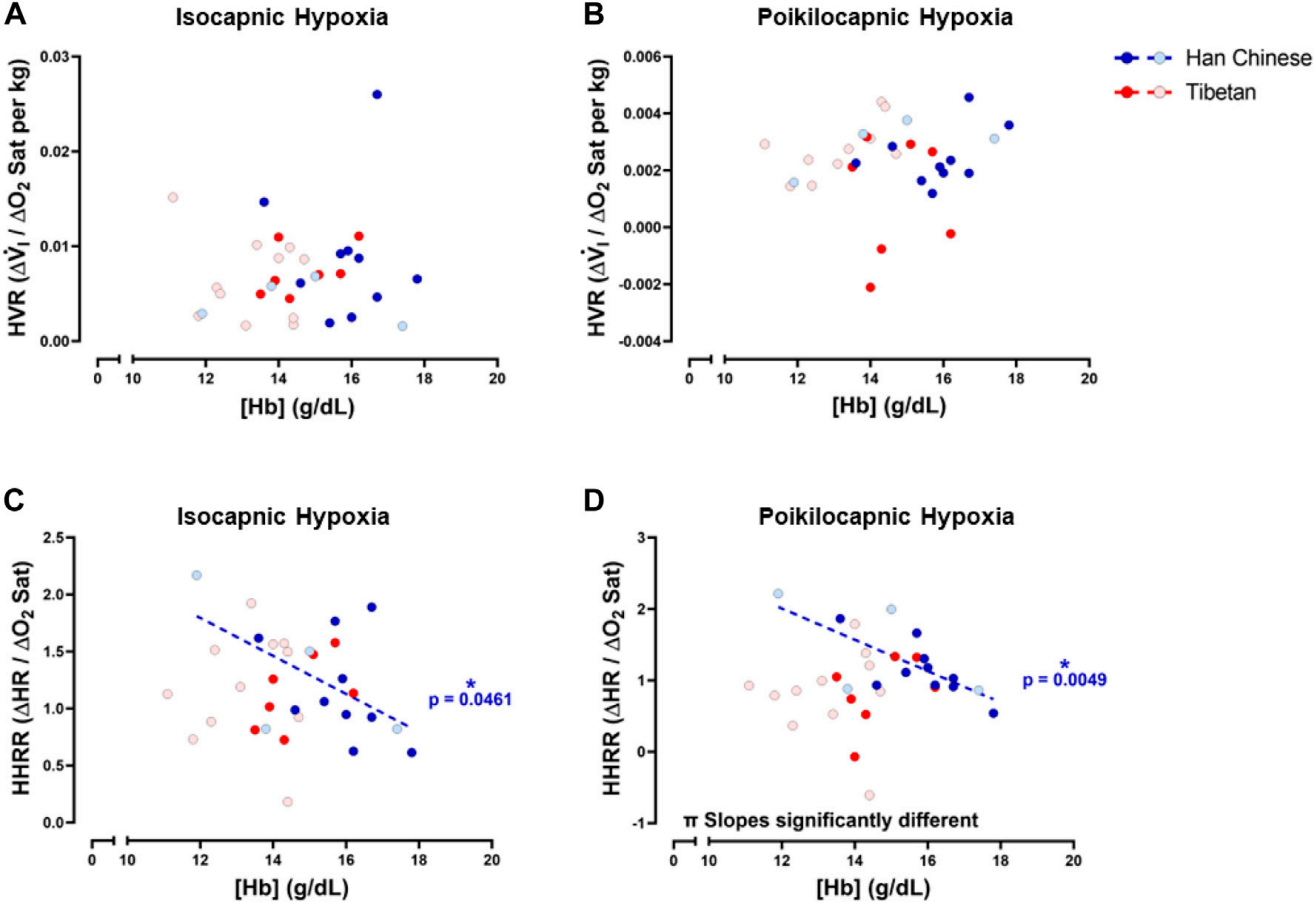
Hemoglobin concentration ([Hb]) *versus* hypoxic ventilatory response (HVR) or hypoxic heart rate response (HHRR) in isocapnic **(A,C)** and poikilocapnic conditions **(B,D)**. Tibetan (red) and Han Chinese (blue), male (dark circles) and female (light circles) individuals. * Significant correlation for Han Chinese (blue asterisk); π *p* < 0.05 comparing slopes for linear regression of Tibetan *versus*. Han Chinese groups. Han Chinese n = 16 (10 males, 6 females); Tibetans n = 20 in isocapnia (8 males, 12 females) and 19 in poikilocapnia (8 males, 11 females).

To study potential relationships of ventilatory and cardiac responses to hypoxia with [Hb], we examined relationships between HVR and HHRR with [Hb] for both isocapnic and poikilocapnic hypoxia. We did not find significant correlations between HVR and [Hb] during isocapnic nor poikilocapnic hypoxia (Figures 8A, B). However, we found significant correlations between HHRR and [Hb] during isocapnic (*p* < 0.05) and poikilocapnic (*p* < 0.005) hypoxia for Han Chinese but not Tibetans (Figures 8C, D); moreover, the slopes of the linear regressions were significantly different between Han Chinese and Tibetans in poikilocapnic hypoxia (Figure 8D; *p* < 0.03). When we performed separated analysis for male and females, we confirmed that relationships between HVR and [Hb] were not significant in male and females, neither for isocapnic nor poikilocapnic hypoxia. The analysis of HHRR *versus* [Hb] separated by sex showed a significant correlation for male Han Chinese during poikilocapnic hypoxia (*p* < 0.02), with no significant correlation for Tibetan males, and slopes were significantly different (*p* < 0.02) between groups.

## Discussion

Our results show Tibetan males exhibit relatively lower [Hb] than Han Chinese males at intermediate altitude, consistent with studies at low and high altitudes, no differences in HVR in contrast to previous findings at high altitude, and novel hypoxia-induced heart rate response differences under poikilocapnic conditions between these groups. Our analyses also revealed differences in the relationship between HVR and HHRR in poikilocapnia and between [Hb] with HHRR in both isocapnic and poikilocapnic conditions.

### Tibetan males exhibit lower [Hb] than Han Chinese males at intermediate altitude

A hallmark phenotype in Tibetans is relatively lower [Hb], which is suggested to be the result of a blunted hematological response to hypoxia (Beall et al., 1998; Petousi and Robbins, 2014; Gassmann et al., 2019), greater plasma volume (Stembridge et al., 2019), or potentially increased red cell destruction (Tift et al., 2020) in this population. Many Tibetans living at high altitude have [Hb] similar to that observed in sea-level lowland residents (Beall et al., 1998) and have lower [Hb] concentrations compared to many adult Han Chinese or Andeans living at similar high altitudes (Garruto et al., 2003; Wu et al., 2005; Beall, 2007). Some high-altitude residents increase [Hb] to pathological levels that are linked to chronic mountain sickness, which has a notably lower prevalence in Tibetans relative to other populations who have lived at high altitude for hundreds of generations (Villafuerte and Corante, 2016; Villafuerte et al., 2022). We found that participants of this study at intermediate altitude exhibited [Hb] within a range similar (Figure 1) to that of lowland residents and Tibetans at high altitude (Beall et al., 1998), which is further consistent with lower [Hb] observed in Tibetans males relative to Han Chinese males living at sea level (Petousi et al., 2014) or Tibetan males and females residing at high altitude (Beall, 2007). Petousi et al. (2014) also measured plasma erythropoietin in Tibetan volunteers exposed to 8 h of hypoxia in a hypobaric chamber and reported the “major Tibetan” allele of *EPAS1* and *EGLN1* genes were associated with a blunted hypoxia-induced increase in plasma erythropoietin when compared to participants with the minor “Tibetan” allele. However, it is also possible that differences in plasma volume (Stembridge et al., 2019) or red blood cell degradation (Tift et al., 2020) may have an effect on different levels of [Hb] measured at low altitude, as has been observed or suggested at high altitude. Thus, independent of the mechanism, it is plausible that differences in [Hb] we observed at intermediate altitude are driven by genetic variants present in the Tibetan population. This is particularly important given that differences in [Hb] vary based on ancestry at different altitudes (Mairbäurl et al., 2020) and sex (Beall, 2007; Gassmann et al., 2019; Mairbäurl et al., 2020); however, most of the studies comparing [Hb] measurements between Han Chinese and Tibetans have been performed in male participants or report values of [Hb] in females at high altitude (Zhuang et al., 1993; Beall, 2007; Gassmann et al., 2019; Mairbäurl et al., 2020). In this study, we report values of [Hb] in women of Tibetan ancestry living at intermediate altitude. We found [Hb] in Tibetan women in this study is similar to those observed in Tibetan women at high altitude (Beall, 2007); however, we did not find significant differences in [Hb] between Tibetan and Han Chinese women. We acknowledge the low number of female individuals studied and that four of these female participants are post-menopausal. Therefore, with this evidence, it is clear that more studies need to be performed in women at sea level and intermediate altitude to determine potential [Hb] differences in based on ancestry, menopausal status, and hormone profile.

### Tibetans and Han Chinese show similar ventilatory responses to isocapnic and poikilocapnic hypoxia at intermediate altitude

We did not find differences in ventilatory parameters during baseline nor acute-hypoxic conditions between Han Chinese and Tibetans. The values of V_T_, f_R,_ and V.i in participants breathing room air as well as the HVRs (both isocapnic and poikilocapnic), and the ventilatory responses to changes in CO_2_ during hypoxia (HCVR), did not show a significant effect of ancestry at intermediate altitude. These findings are different from those reported for Han Chinese and Tibetans residing at high altitude (Zhuang et al., 1993; Curran et al., 1997), although they agree partially with data from Sherpas compared to “Westerners” at intermediate and high altitude (Hackett et al., 1980) and Tibetans *versus* Han Chinese at sea level (Petousi et al., 2014). Zhuang et al. compared lifelong Tibetan residents with acclimatized Han Chinese newcomers at 3,658 m and described increased minute ventilation breathing room air and increased hypoxic sensitivity (parameter A from the HVR, with hyperbolic adjustment) for the Tibetan group under isocapnic conditions. However, they did not find differences between groups when HVR values of delta V. i over delta Sp_O2_ were compared. Despite differences in hypoxic sensitivity, the authors compared their measurements with others published previously and found that hypoxic sensitivity in high-altitude Tibetans was similar to low-altitude residents or acclimatized newcomers, demonstrating that Tibetans do not exhibit a blunted HVR and, on the contrary, they maintain increased ventilation as if they just arrived to high-altitude (Zhuang et al., 1993). Petousi et al. (2014) studied Han Chinese and Tibetan participants residing at sea level and found that Tibetans had lower End-tidal PetCO_2_ values than Han Chinese (indicating increased baseline ventilation for a given metabolic rate), but the authors also found similar values of ventilation in normoxia and acute hypoxic ventilatory sensitivity during isocapnia for Han Chinese and Tibetans (Petousi et al., 2014). Hence, differences in End-tidal PetCO_2_ must reflect metabolic differences, as well as potential differences in CO_2_ regulation. A study performed in Sherpas and “Westerners” at low and high altitude demonstrated higher values of resting ventilation in the Sherpa group, although they found that HVR values were not different between groups when corrected by body size (Hackett et al., 1980).

Thus, our results at intermediate altitude agree with previous literature observing similar acute ventilatory responses between Han Chinese and Tibetans at sea level and high altitude, although we did not find differences in baseline ventilation based on analyses of both male and female participants. Although our analysis did not reveal sex and/or ancestry effects for End-tidal PetCO_2_ nor HVR, it will be necessary to perform a large-scale analysis regarding sex differences in these populations. It is plausible that the severity of hypoxia is not enough to produce significant differences in resting ventilation between Han Chinese and Tibetans or that such differences are not present in the absence of long-term high-altitude exposure.

### Tibetans show blunted heart rate responses to hypoxia

Exposure to acute hypoxia produced the expected increase in heart rate, which stabilized during 5 minutes of the stimulus. We found the HHRR was similar during isocapnic hypoxia between ancestry groups; however, during poikilocapnic hypoxia, the increase of HR was smaller in the Tibetans compared to the average Han Chinese response (Figure 4D). Moreover, when we quantified the heart rate responses to changes in CO_2_ during hypoxia (or HCHRR), we found a significant difference with an average negative value in Tibetans contrasting the positive average value for the Han Chinese group (Figure 5D). These results highlight an inhibitory effect of lower values of PetCO_2_ (due to hyperventilation when we do not control CO_2_) on heart rate that is present in the Tibetan group but not in the Han Chinese. To our knowledge, this is the first time that population-related differences in HR response to hypoxia are found in people not living in extreme high-altitude conditions. The range of values of HHRR that we observed for Han Chinese (0.9–1.28) is comparable to those reported before for individuals from sea-level populations after 5 min of isocapnic and poikilocapnic hypoxia in healthy men (Simon et al., 1995; Ainslie and Poulin, 2004; Steinback and Poulin, 2008). Experiments studying the mechanisms of HVR establish that the acute ventilatory response is mainly attributed to activation of the peripheral chemoreflex starting in the carotid bodies, the cardiac and vascular changes induced by hypoxia are influenced by more than one process. The cardiovascular responses to hypoxia occur by activation of several mechanism including: stimulation of central nervous system and arterial chemoreceptors, direct effects of hypoxia and hypocapnia on heart and vasculature, modulation of the nervous system by secondary mechanisms such as pulmonary stretch afferents, alkalosis produced by hyperventilation, and increased secretion of catecholamine from the adrenal glands (de Burgh Daly, 1997; Joyce and Wang, 2022). In particular, the changes of heart rate observed in acute hypoxia are the result of activation of sympathetic and vagal cardiac efferent activities that can be triggered by the secondary inputs mentioned (de Burgh Daly, 1997). Studies performed in dogs and humans concluded that the primary HR response resulting from isolated activation of the carotid bodies is bradycardia (Downing et al., 1962; de Burgh Daly, 1997; de Burgh Daly et al., 2000; Burgh Daly, 2011), and patients with resected carotid bodies show tachycardia with breath-holding in an oxygen-tension-dependent way (Gross et al., 1976). With this, we initially speculated that Tibetans may have increased arterial chemoreceptor activity resulting in smaller HR responses during hypoxia. However, the fact that we did not find differences in ventilatory parameters (Figure 3) nor in Dejour’s test (data not shown) associated with ancestry suggests that the blunted heart rate responses observed in Tibetans in poikilocapnic conditions (Figure 4D; Figure 5D; Figure 7B) are regulated by differential effects to CO_2_ sensitivity more than O_2_-derived mechanisms. Our finding of blunted heart rate responses in Tibetans during poikilocapnia, but not isocapnia, reflects a realistic situation of hypoxia exposure where hyperventilation produces a decrease in PetCO_2_ and highlights the role central CO_2_ chemoreceptors may have in the differential cardiovascular responses to hypoxia in populations historically living at high altitude. When we compared the HR response in poikilocapnic *versus* isocapnic hypoxia, Han Chinese showed a positive value of HCHRR, reflecting that the average HR in poikilocapnic hypoxia is higher than in isocapnic hypoxia (Figures 5C, D). Early reports found similar results, with smaller heart rate responses to hypoxia in conditions of isocapnia when compared to poikilocapnic hypoxia (Richardson et al., 1966). However, we found for the first time that Tibetans have different HR responses with an average negative HCHRR value, meaning that, on average, Tibetans have lower HR during poikilocapnic hypoxia compared to isocapnic hypoxia (Figures 5C, D). This finding in Tibetans who are not exposed to high altitude suggests that genetic factors, such as those previously reported, including those in the hypoxia inducible factor pathway (Simonson, 2015; Yu et al., 2022; Lawrence et al., 2024), may preserve high-altitude features in these populations even when living closer to normoxic conditions.

Exposure to acute hypoxia produces an increase of HR concomitant to an increased sympathetic activity (Halliwill et al., 2003; Hanada et al., 2003), and in high-altitude hypoxia, the increase in heart rate is related to an increase in sympathetic activity and vagal withdrawal (Koller et al., 1988; Hansen and Sander, 2003; Bärtsch and Gibbs, 2007). Thus, it is also possible that genetic factors determining different autonomic responses to hypoxia in human populations explain different HR responses that we observed at intermediate altitude, with lower HHRR and HCHRR in Tibetans compared to Han Chinese groups. In fact, increased sympathetic activity is observed in both lowlanders and Andeans exposed to high altitude (Hansen and Sander, 2003; Lundby et al., 2018; Simpson et al., 2021a; Simpson et al., 2021b), whereas Sherpa highlanders show lower muscle sympathetic nerve activity at high altitude (Simpson et al., 2019; Simpson et al., 2021b). The differences in the autonomic responses between lowlanders and/or Andeans and Sherpa populations could also be explained by genetic factors contributing to the differential responses observed in Tibetans. Zhou et al. studied Tibetans born and raised at high altitude (3700 m) who migrated to sea level for four years and compared them with a lowland Han Chinese group. The authors exposed both groups to acute hypoxia in a hypobaric chamber (2 h, simulating 3700 m) and found that acute hypoxia induced a significant increase of HR in the Han Chinese but not in the Tibetan population. They attribute these differences to changes in sympathetic stimulation during acute hypoxia (based on heart rate variability parameters) that occur in Han Chinese but were not observed in the Tibetan group (Zhou et al., 2008). These responses may manifest also at lower altitudes in individuals with high-altitude ancestry. For example, Petousi et al. found blunted pulmonary vascular responses to acute isocapnic hypoxia in a Tibetan population living at sea level when compared to a Han Chinese population residing in the same location (Petousi et al., 2014). Another argument to explain the observed differences in HHRR may be distinct values of stroke volume in Tibetan and Han Chinese populations that compensate for differences in heart rate during hypoxia. Reports studying Tibetan and Han Chinese individuals have shown similar stroke volume and cardiac output in resting conditions at more than 3417 m of altitude (Ge et al., 1995; Chen et al., 1997; Simonson et al., 2015), at sea level, and during maximum exercise (Simonson et al., 2015). Petousi et al. did not find significant differences in the cardiac output of Tibetan and Han Chinese in resting conditions, either during normoxia nor during isocapnic hypoxia (Petousi et al., 2014), but the changes of cardiac output during poikilocapnic hypoxia have not been studied. Thus, more studies are required to establish the responses to acute hypoxia in these different populations and which high-altitude phenotypes are maintained under sea-level conditions.

### Tibetans and Han Chinese have different Integrated ventilatory and heart rate responses to hypoxia during poikilocapnic hypoxia

In order to characterize phenotypic differences between Tibetan and Han Chinese groups during hypoxia, we studied the correlations between HVR and HHRR and also between these parameters and [Hb]. We acknowledge that correlations are not a mechanistic approach to study causal mechanisms of the potential genetic or CO_2_-dependent contributors to the responses to hypoxia. However, these observations in human populations may provide insight into potential mechanisms that could be studied in animal or cellular models that help to elucidate relevance in evolutionary contexts and/or hypoxia-related pathological conditions.

The mechanisms involved in control of breathing and heart rate are considered independent, although these two variables can interact, for example, during respiratory sinus arrhythmia (Perry et al., 2019; Hayano et al., 1996). A high level of correlation is described between minute ventilation and HR, particularly during aerobic work (Vai et al., 1988; Treese et al., 1993; Mermier et al., 1993) with both V.i and HR positively correlated with oxygen uptake (Gastinger et al., 2010). Richalet and Lhuissier proposed that during moderate exercise, HVR and HHRR exhibit a negative association in sojourners exposed to high altitude (Richalet and Lhuissier, 2015) and therefore poikilocapnic hypoxia. Our study is the first to show these correlations in Han Chinese and Tibetan groups. We found that Tibetans have a positive correlation of HVR and HHRR in poikilocapnic hypoxia, with a slope significantly different than the Han Chinese group. The Han Chinese group shows a negative slope although the correlation of HVR and HHRR was not significant in our results. Future studies with a larger number of participants need to be performed to determine if people with Han Chinese ancestry show negative correlations of HVR and HHRR.

The correlation between ventilatory and heart rate responses to hypoxia also varies across species (Burgh Daly, 2011). Stimulation of arterial chemoreceptors with hypoxic blood produced increased ventilation in various species but produced bradycardia in seals and cats (Macleod and Scott, 1964; Elsner et al., 1977), a mixed bradycardic and tachycardic response in dogs (de Burgh Daly and Scott, 1958), and complete tachycardia in monkeys (de Burgh Daly et al., 1978; Burgh Daly, 2011). The correlation between ventilation and heart rate response induced in hypoxia in humans is similar to the one found in primates, with tachycardia observed concomitant to increased ventilation, although a significant correlation of HVR *versus* HHRR has not been found in conditions of isocapnic hypoxia (Slutsky and Rebuck, 1978; Simon et al., 1995). Our results agree with these results showing that, in isocapnic hypoxia, there is no correlation between HVR and HHRR, and we did not find differences between linear regressions of Tibetans and Han Chinese (Figure 7A). However, during poikilocapnic hypoxia, we found a positive significant correlation in Tibetans that was not observed in the Han Chinese group, and the slopes of the Tibetan and Han Chinese group were significantly different (Figure 7B). Thus, there is an effect of lower CO_2_ due to hyperventilation that is affecting Tibetan and Han Chinese populations differently. This effect must be attributed to CO_2_ since these dissimilar relationships are not observed in isocapnic conditions, thus affecting this correlation due to different HR responses in poikilocapnia.

We found negative associations between [Hb] and HHRR in Han Chinese in both hypoxic isocapnic and poikilocapnic conditions (Figures 8C, D). A similar negative correlation is also observed in individuals with excessive erythrocytosis and blunted HHRR in isocapnic hypoxia in a study that included Andean participants with chronic mountain sickness (Heinrich et al., 2020). We found these negative [Hb] *versus* HHRR associations even when none of our Han Chinese participants showed levels of [Hb] outside the range expected at sea level. On the other hand, we did not find significant correlations of [Hb] *versus* HHRR in our Tibetan group (Figure 8). These profiles of correlations between [Hb] and HHRR for Han Chinese and Tibetans are independent of the isocapnic or poikilocapnic status; therefore, these differences are attributed to ancestry but not to an inhibitory effect of CO_2_. Recent studies indicate [Hb] increases consistently with altitude across several human populations, but the increase in [Hb] with altitude is greater only among individuals of native South American ancestry (Gassmann et al., 2019) *versus* a reference group. In the same manuscript, in Tibetans, the correlation between [Hb] and altitude is significant, but not different from the reference group, highlighting the potential for distinct physiological outcomes such as the prevalence of chronic mountain sickness, which is much less common among Tibetans (Gassman et al., 2019). Whether excessive erythrocytosis and potential deleterious effects of increased blood viscosity are absent in Tibetans or if there are different mechanisms modulating vascular contractility to reduce vascular resistance during hypoxia remains to be determined.

The main limitation of this study is a limited number of participants, which are similar to other studies of ventilatory responses to hypoxia (Zhang and Robbins, 2000; Brutsaert et al., 2005). However, in this study, we examine ventilatory and heart rate responses in both males and females, four of whom were post-menopausal. Future studies that aim to understand sex differences in physiological responses to hypoxia and the contribution of hormones and menopausal status to ventilatory responses to hypoxia are needed in populations with a history of high-altitude residence.

## Conclusion

The average lower [Hb] observed in Tibetan males confirm a phenotype associated (directly or indirectly) with high-altitude adaptation that is maintained at intermediate altitude. The hypoxic ventilatory responses in Tibetans living at intermediate altitude were not different from Han Chinese living in the same location during isocapnic or poikilocapnic conditions. Compared to Han Chinese, Tibetans exhibited attenuated heart rate responses to hypoxia in poikilocapnic conditions, whereas isocapnic responses were not different. Also, the relationship between HVR and HHRR was different during poikilocapnia but not in isocapnia, highlighting the differential effect of CO_2_ on the coordinated breathing and heart rate responses to hypoxia in people with Tibetan ancestry. The potential contribution of molecular signals to high-altitude hypoxic adaptation as well as the physiological responses driven by these pathways when living at different altitudes require further investigation.

## Data Availability

The raw data supporting the conclusion of this article will be made available by the authors, without undue reservation.
